# Sorting cells of the microalga *Chlorococcum littorale* with increased triacylglycerol productivity

**DOI:** 10.1186/s13068-016-0595-x

**Published:** 2016-08-31

**Authors:** Iago Teles Dominguez Cabanelas, Mathijs van der Zwart, Dorinde M. M. Kleinegris, René H. Wijffels, Maria J. Barbosa

**Affiliations:** 1 Wageningen UR, Bioprocess Engineering, AlgaePARC, P.O. Box 16, 6700 AA Wageningen, The Netherlands; 2 Wageningen UR, Food and Biobased Research, AlgaePARC, Wageningen, The Netherlands; 3 Wageningen UR, Bioprocess Engineering, Wageningen, The Netherlands; 4 Faculty Biosciences and Aquaculture, Nord University, Bodø, Norway

**Keywords:** Microalgae, *Chlorococcum littorale*, Fluorescence assisted cell sorting (FACS), Strain improvement, Lipid productivity

## Abstract

**Background:**

Despite extensive research in the last decades, microalgae are still only economically feasible for high valued markets. Strain improvement is a strategy to increase productivities, hence reducing costs. In this work, we focus on microalgae selection: taking advantage of the natural biological variability of species to select variations based on desired characteristics. We focused on triacylglycerol (TAG), which have applications ranging from biodiesel to high-value omega-3 fatty-acids. Hence, we demonstrated a strategy to sort microalgae cells with increased TAG productivity.

**Results:**

1. We successfully identified sub-populations of cells with increased TAG productivity using Fluorescence assisted cell sorting (FACS). 2. We sequentially sorted cells after repeated cycles of N-starvation, resulting in five sorted populations (S1–S5). 3. The comparison between sorted and original populations showed that S5 had the highest TAG productivity [0.34 against 0.18 g l^−1^ day^−1^ (original), continuous light]. 4. Original and S5 were compared in lab-scale reactors under simulated summer conditions confirming the increased TAG productivity of S5 (0.4 against 0.2 g l^−1^ day^−1^). Biomass composition analyses showed that S5 produced more biomass under N-starvation because of an increase only in TAG content and, flow cytometry showed that our selection removed cells with lower efficiency in producing TAGs.

**Conclusions:**

All combined, our results present a successful strategy to improve the TAG productivity of *Chlorococcum littorale*, without resourcing to genetic manipulation or random mutagenesis. Additionally, the improved TAG productivity of S5 was confirmed under simulated summer conditions, highlighting the industrial potential of S5 for microalgal TAG production.

**Graphical abstract:**

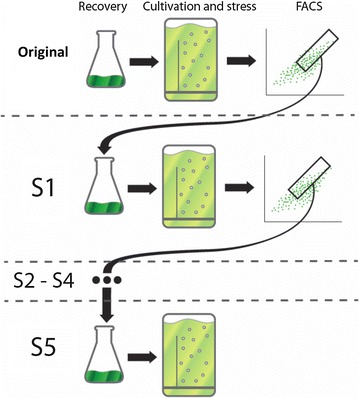

## Background

Microalgae are a versatile feedstock with applications ranging from animal feed to high-end products like nutraceuticals [1]. Economic analyses show production costs from 5–6€ per kg of dry biomass [2], which clearly require further reduction if aiming at commodities market. Despite extensive research in the last decades, microalgae are still only economically feasible for high valued markets, e.g. nutraceuticals and also as feed for aquaculture [3–5].

Cost reduction can be achieved by two ways: (1) decreasing capital and operational costs of the cultivation systems) and (2) increasing microalgae productivity (thus increasing the yields on light). The yields can be increased via process optimization to improve the light usage by the cells [6] or via developing new strains with superior performance [7]. Strain improvement has multiple approaches that could be used: selection of cells [8], adaptive laboratory evolution [9], random mutagenesis [10] and genetic manipulation [7, 11].

In this work, we focus on microalgae selection: taking advantage of the natural biological variability of species to select variations based on desired characteristics [7]. Microalgae are microscopic phototrophs with unicellular species ranging from 1 to 30 µm in size [12]. Due to their size we need a technology that can quickly analyze and select cells of interest. Fluorescence activated cell sorting (FACS) is a well-established technology that has been used since the 1970s for single cell analyses of mammalian and plant cells. A few research groups have recently applied FACS to microalgae with different approaches and outcomes, mainly focusing on selecting lipid-rich cells [8, 13, 14]. Most of the works on FACS to improve lipid content of microalgae strains present also a combination with different mutagenesis techniques [7, 10, 15, 16].

All above mentioned articles, however, presented results that were carried out in small volumes (well-plates or shake flasks) and do not refer to biomass productivity or growth rates of the sorted/improved strains. Hence, estimation of industrial performance of strains is limited. The exception was the work of Beacham et al. [15], that reported a reduction in biomass productivity in all high-lipid sorted/mutated strains, resulting in only one sorted/mutant cell line with increased lipid productivity when compared to their original culture. Another work reported a 3.2 times slower growth rate in their high-lipid sorted strain [14]. Clearly, using FACS (with or without mutagenesis) to select lipid-rich cells may lead to undesirable results to cell growth, which would affect directly lipid productivity. Hence, it is necessary to evaluate the effect of the sorting on biomass/product productivity to assess the industrial potential of developed strains.

In this work, we present a strategy to find and sort microalgae cells with increased TAG productivity. Our approach explores the natural variation of microalgae (no mutagenesis involved). Additionally, our approach evaluated the industrial potential of the selected population, evaluating the performance under simulated summer conditions. Our approach was developed in the following steps: (1) to analyze cell properties at population level to identify sub-populations with potentially increased TAG productivity; (2) to sequentially sort (5x) cells under N-starvation as a selective pressure towards increased TAG productivities; (3) to confirm the increased lipid productivity of our sorted populations with lab-scale photobioreactors experiments, to measure biomass accumulation and also biomass composition; (4) the sorted population with the highest lipid productivity (S5) was run in comparison with the original strain in an indoor photobioreactor under simulated Dutch summer conditions.

## Methods

### Inoculum preparation, cultivation and culture screening with FACS

*Chlorococcum littorale* (NBRC 102761) was used as the original strain. Inocula from the original strain was prepared from samples preserved in petri dishes containing growth medium and agar (12 g l^−1^), under low light conditions (16 µmol m^−2^ s^−1^). Homogenous samples were taken from agar to 200 ml sterile borosilicate Erlenmeyer flasks, containing 100 ml of sterile growth medium. Artificial seawater was used as grown medium with the following composition (g l^−1^): NaCl 24.55, MgSO_4_·7H_2_O 6.60, MgCl_2_·6H_2_O 5.60, CaCl_2_·2H_2_O 1.50, NaNO_3_ 1.70, HEPES 11.92, NaHCO_3_ 0.84, EDTA-Fe(III) 4.28, K_2_HPO_4_ 0.13, KH_2_PO_4_ 0.04. Additionally, the medium contained the following trace elements (mg l^−1^): Na_2_EDTA·2H_2_O 0.19, ZnSO_4_·7H_2_O 0.022, CoCl·6H_2_O 0.01, MnCl_2_·2H_2_O 0.148, Na_2_MoO_4_·2H_2_O 0.06, CuSO_4_·5H_2_O 0.01.

Initially, N-starved cells (using the experimental set-up as shown in 0) of *C. littorale* were used to analyze cell properties at population level. The population analyses were done to choose the boundaries of the sorting gate. Samples were run in a FACS Calibur^®^ to measure autofluorescence (FL3, 670 nm LP), lipid fluorescence (FL1, 530/30 nm) and forward scatter (full settings given at “Cell sorting using FACS”). FlowCAM^®^ fluid imaging was used to complement the assessment of the population characteristics, providing the actual cell diameter (µm) and photomicrographs of the cells (the same parameters as the FACS were also measured, autofluorescence and lipid fluorescence). The lipid fluorescence was measured using the Bodipy 505/515 (BP) stain following the protocol developed previously [17].

### Experimental set-up

For the N-runout/sorting experiments *C. littorale* was cultivated in a 0.4 L flat panel photobioreactor under controlled and sterile conditions (25 °C, air flow at 1 v/v/m, pH set at 7.0 controlled on demand via CO_2_ addition to air, constant light at 400 µmols m^2^ s^−1^), specifications of the photobioreactor can be found at Breuer et al. [18]. The cultivations were nitrogen run-out batches, i.e. nitrogen was added at an initial concentration of 10.7 mM (as KNO_3_) and was allowed to be taken up by the cells. All nitrogen was consumed within the first 48 h, after which the N-starvation phase is considered. At the end of the nitrogen starvation phase (after 8 days of cultivation), when the cells reached the maximum lipid-fluorescence, a sample was taken to the FACSCalibur to be measured and sorted. At each sorting round 1000 cells were sorted into a sterile falcon tube (50 ml) and subsequently centrifuged (1050×*g*, 10 min) and transferred to a new sterile falcon tube containing 20 ml of fresh sterile medium.

The sorted cells were allowed to recover and grow under low light conditions for 2 weeks (constant at 16 µmols m^2^ s^−1^). After 2 weeks of growth the cells were transferred to a sterile Erlenmeyer flask containing 100 ml of fresh sterile medium, to continue the cycles of starvation/sorting. The algae were grown in a INFORS shake incubator under controlled constant conditions for 2 weeks (25 C, 120 rpm, 50 % air moist and 120 µmols m^2^ s^−1^). After 2 weeks in the incubator the cells were used as inoculum to re-start the N-runout/sorting procedure. Additionally, a sample of the sorted populations was plated on agar plates (with N replete medium) for long term preservation (refreshed every 3 months). Additionally, samples from the sorted populations were plated in agar plates for storage and for producing inocula for the comparison experiment.

A second experiment was done to compare the sorted populations with the original population in parallel, using the same experimental set-up as described above. The original population was compared with 4 different sorted population (S2–S5; the numbers refer to the round of N-starvation/sorting). The first sorted population (S1) was kept out of this experiment because it showed no differences when compared with the original population in the first experiment (data not shown). For this new experiments all the different populations (original, S2–S5) were transferred from agar plates to Erlenmeyer flask and next to photobioreactors and cultivated under the similar conditions as above mentioned. Biomass samples from each reactor were taken at the end of the N-starvation phase for analyzing the biochemical composition (as showed below at “Biomass analyses”).

A final experiment was conducted to compare the original with the S5, which was the population with the highest TAG productivity in the previous experiments. The goal of the experiment was to compare both populations under simulated Dutch summer conditions (day length of 16 h), hence the light was supplied with an sinus function with a solar noon at 1500 µmols m^2^ s^−1^ supplied as a day/night cycle (resulting in an average daily incident light intensity of 636 µmols m^2^ s^−1^). This experiment was done in a flat panel reactor with a working volume of 1.9 L, light path of 0.02 m, and 0.08 m^2^ surface area (Labfors, Infors HT, 2010). Air flow as set at 1.0 l min^−1^ and mixed with CO_2_ on demand to keep the pH constant at 7.0. Temperature was kept constant with an integrated water-jacket at 25 °C.

### Daily measurements

Daily samples were taken always at the same time. Optical densities were used as a proxy for cell density (750 nm) and chlorophyll (680 nm) using a spectrophotometer (HACH, DR5000) [19]. FlowCAM^®^ fluidic imagining was daily used to follow-up the cell’s diameter, autofluorescence (chlorophyll fluorescence) and lipid-dependent fluorescence (BODIPY_505/515_ fluorescence). Samples were diluted to meet a PPUI (particle per use image) between 1.0 and 1.2 and then stained for neutral lipids using BODIPY_505/515_ at 0.4 µg/ml diluted in 0.35 % ethanol [17]. After 5 min of dark incubation the samples were run in the FlowCAM^®^ using the following settings: 20× optical magnification and Trigger-mode-on based on the fluorescence of channels 1 (autofluorescence, 650 nm long pass filter) channel 2 (BODIPY_505/515_, 525/30 nm filter).

Samples were also taken for analyses of extracellular nitrogen content (N-NO_3_ mg l^−1^). Hence, 1 ml of culture was centrifuged at 13.000×*g* and the supernatant was separated to be analyzed. Sulphanilamide N-1-naphthyl method was used (APHA 4500-NO3-F) by an SEAL AQ2 automatic analyzer.

### Cell sorting using FACS

FACSCalibur^®^ (BD life technologies) was used to measure cell properties and to sort cells. The measurements were lipid-dependent fluorescence (Bodipy 505/515, FL1 channel) and chlorophyll-dependent (autofluorescence, FL3 channel). Both channels were measured at log scale and the sensitivity was set at 300 mV. All samples were diluted to a concentration of 200 events/second (OD_750_ of approximately 0.1). Measurements of 10,000 and 1000 cell’s per sample were saved and further used for analyses. 1000 cells were sorted and used in the experiments.

**Fig. 1:**
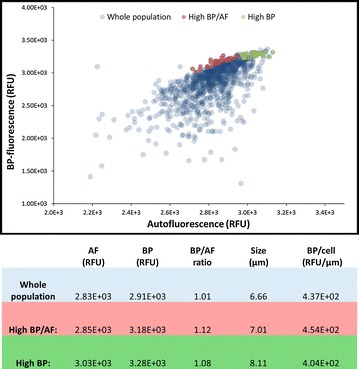
Flow cytometry data can identify high-lipid content cells among the population. The *graph* shows a *scatter-plot* between lipid fluorescence (Bodipy, BP; *y*-axis) and chlorophyll fluorescence (Autofluorescence, AF, *x*-axis), in which we can see the whole population (in *blue*) and two sub-populations marked as relevant: cells with the highest values of lipid fluorescence (high BP; in *green*), and cells that show the highest ratio between lipid and chlorophyll (high BP/AF; in *red*). The *colored* sections in the graph correspond to the *rows* with the *same color* in the table. The *table* show the median values of AF, BP, cell size and the ratios between median values of BP/AF and BP/cell

Sorting was done using the same settings as above, using the gate depicted in Fig. 1. The system was fluxed for 10 min with ethanol 70 % to reduce contamination. Autoclaved and filtered (0.2 µm) PBS was used as sheath fluid (phosphate saline buffer (1 × PBS); composition: NaCl 137 mM, KCl 2.7 mM, Na_2_HPO_4_ 10.0 mM, KH_2_PO_4_ 1.8 mM).

Cells were centrifuged immediately after the sorting (1224×*g* for 5 min) and re-suspended in sterile growth medium. The tubes containing sorted cells were placed under constant light at 16 μmol m^−2^ s^−1^, allowing cells to recover and grow for 2 weeks. The tubes were transferred to flasks, after the growth time, to make inoculum for the next round of N-runout/sorting.

### Biomass analyses

Biomass samples were collected at the end of the N-runout for composition analysis, all results are expressed as  %DW. Biomass was centrifuged twice and washed with MilliQ water (3134×*g* for 10 min at 4 °C) and followed by freezing at −20 °C and freeze-drying for 24 h.

Polar lipids and triacylglycerol’s (TAGs) were extracted and quantified using GC/MS column chromatography of the chloroform fraction of lipids, described by Breuer et al. [18]. Total carbohydrates were analyzed with the phenol–sulfuric acid method developed by Dubois et al. [20]. Total starch was analyzed after extraction with 80 % ethanol followed by quantification using the colorimetric/enzymatic method with the commercial kit from Megazyme (K-STA kit, UK). Total proteins were measured using the colorimetric method developed by Bradford [21].

### Data analysis

Cell analyses from the FlowCAM were exported to Microsoft Excel to plot graphs and estimate descriptive statistics. Flow cytometry data (FSC files) were analyzed with the software FlowJO v 10. Null hypothesis significance testing (NHST) was used to compare the results between and among samples. One-way analysis of variance (ANOVA) was used to access the significance of differences between different groups (SigmaPlot, v. 12.5). The premises of ANOVA, i.e. the homogeneity of variances and the normality of the data, were also measured with SigmaPlot (for all testes *α* was set at 0.05). For population-wide data (flow cytometry) the large data set (10,000 measurements per sample) was compared using size-independent parameters, i.e. the median and the coefficient of variation (CV). Confidence intervals (CI) and histogram overlapping were used to compare populations.

### Calculations

The biomass dry weight was used to calculate the growth rate (µ, day^−1^), as given in (Eq. 1),

1 $$\upmu = \frac{{{ \ln }\left( {{\text{DW}}_{{t_{\text{final}} }} - {\text{DW}}_{{t_{0} }} } \right)}}{{t_{\text{final}} - t_{0} }},$$

where, DW stands for the dry weight of biomass (g l^−1^), from the first (*t*_0_) and the last time point (*t*_final_) considered. For the growth rate under nitrogen replete conditions (N+) the 2nd day of cultivation (*d* = 2) and the day of inoculation (*d* = 0) were used as final and initial values, respectively.

Relative lipid production was measured by BODIPY cellular fluorescence to calculate the BODIPY accumulation rate (BP_*r*_), relating to the increase of cellular neutral lipids in time. The BP_*r*_ was estimated with an approach similar to Eq. 1, however the values of DW where substituted with the values of median BP fluorescence. The time interval used for calculation was the period between the start (*d* = 2) and the end (*d* = 8) of the nitrogen run-out.

The volumetric biomass productivity during the growth phase (N+; *P*_*X*_, g l^−1^ day^−1^) was estimated with (Eq. 2),

2 $$P_{X} = \frac{{{\text{DW}}_{{t_{\text{f}} }} - {\text{DW}}_{{t_{0} }} }}{{t_{\text{f}} - t_{0} }},$$

where, the values of dry weight (DW, in g per liter) were used between the end (*t*_f_ = 2) and the beginning (*t*_0_ = 0) of the growth phase (for both experiments as presented in 1.2 and 1.3).

The maximum average TAG volumetric productivity (P_TAG_, g l^−1^ day^−1^) was estimated using (Eq. 3),

3 $$P_{\text{TAG}} = \frac{{{\text{TAG}}_{\text{f}} - {\text{TAG}}_{0} }}{{t_{\text{f}} - t_{0} }},$$

where, TAG_f_ is the TAG concentration at the end of the production period (in g l^−1^) and TAG_0_ is the TAG concentration at the start of the N-starvation (also in g l^−1^). The corresponding time period was represented by the final and initial days of TAG production (respectively, *t*_f_ and *t*_0_). For the first experiment (“Sorting lipid-rich cells”), we used the total N-starvation period, hence between *t* = 8 and *t* = 2. For the second experiment (“Comparing original and S5 under simulated Dutch summer conditions”), comparing original and S5, we used the period which showed the maximum TAG productivity, hence between *t* = 4 and *t* = 2.

TAG yields (g mol^−1^) on light were calculated using the areal TAG productivity (g m^2^ day^−1^) divided by the corresponding total amount of light impinging on the surface of the reactor (in mol m^2^ day^−1^).

## Results and discussion

### Gate set-up to sort lipid-rich cells

Our approach started by choosing the sorting criteria. Besides the obvious choice of lipid-dependent fluorescence (Bodipy, BP) we also included chlorophyll-dependent fluorescence (Autofluorescence, AF). We included AF for two reasons: the first reason is because AF correlates to cell size, hence it can be used to estimate the ratio lipid/cell. Secondly, we hypothesized that cells that could keep their levels of AF after a period of N-starvation would be cells with low levels of chlorophyll degradation, hence cells that could grow again if N is re-supplied. We were surprised to observe an almost absence of chlorophyll fluorescence degradation after long periods of N starvation. This has been observed in our previous publication [22] and might be due to the fact that *C. littorale* is a highly resilient strain under abiotic stress [23–25].

To establish the sorting gate we first carried out a screening test in which N-starved cells (under the same conditions to be used in the experiments to follow) were analyzed with flow cytometry. The scatter-plot between autofluorescence (chlorophyll-dependent, AF) and Bodipy fluorescence (lipid-dependent, BP) (Fig. 1) shows that there are some regions of the population in which these two variables correlate more. Hence, we included the BP/AF ratio as a criterion to estimate the amount of lipids (BP) per cell size (AF). Our analyses of the population of N-starved cells pointed to two interesting sub-populations, as showed in Fig. 1.

The first sub-population to be analyzed were the cells that presented the highest values of lipid fluorescence (High BP). These cells, however, show high values of BP because they are also the biggest cells within the population, which can be evidenced by its bigger size in comparison with the whole population (8.11 against 6.66 µm; Table in Fig. 1). Cell size could be a misleading variable since bigger cells might have, proportionally, the same lipid content as the population’s median. When normalizing the median BP fluorescence by the median cell volume of each population it is clear that the sub-population marked as High BP doesn’t have more fluorescence per cell volume than the whole population (4.04 against 4.37E+03 BP/cell; Fig. 1).

The other sub-population was chosen using the BP/AF ratio as criterion (High BP/AF, Fig. 1). We hypothesized that BP/AF ratio would lead to a sub-population of cells with a higher lipid content per cell. At the same time this sub-population selects the top lipid producers while keeping the median AF value, hence avoiding the AF as a cofounding variable (either due to cell size or relative chlorophyll content). The normalized BP/cell volume show us that indeed the High BP/AF sub-population has a higher value of BP/cell volume in comparison with the whole population (4.54 versus 4.37E+03, Fig. 1). Thus, the gate depicted in red on Fig. 1 as BP/AF was chosen as sorting criteria.

### Sorting lipid-rich cells

The sorting’s were done as depicted in the graphical abstract. After each round of sorting the cells were transferred to shake flasks to produce new inoculum to start a new N-runout (and consequently a new round of sorting). Additionally, an aliquot of the sorted cells was transferred to an agar plate and kept under low light conditions for long term storage and further use for comparing all sorted populations. All sorting rounds had a period of 1 month in between to grow the inoculum after sorting. Hence, each new round of N-runout was started with cells that came from different acclimation conditions than the Wt. Therefore, we produced inoculum from original and all sorted populations that were kept on plate, at the same time and started parallel runs with an independent reactor for each population. The population from the first round of sorting (S1) was kept out of the parallel runs because preliminary data from flow cytometry showed that the cells of S1 had the same distribution of lipid fluorescence as the original (data not shown).

**Fig. 2:**
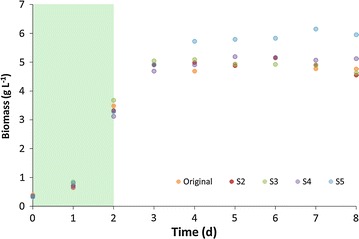
All sorted populations are similar to the wildtype (Wt) in the growth phase. The *graphs* shows the biomass concentration per day (g l^−1^). The *green* area (days 0–2) marks the growth phase, hence both populations consumed nitrogen at the same rate. After day 2 all nitrogen was taken up by the cells. From day 2 onwards it should be considered as starvation phase (N−)

**Fig. 3:**
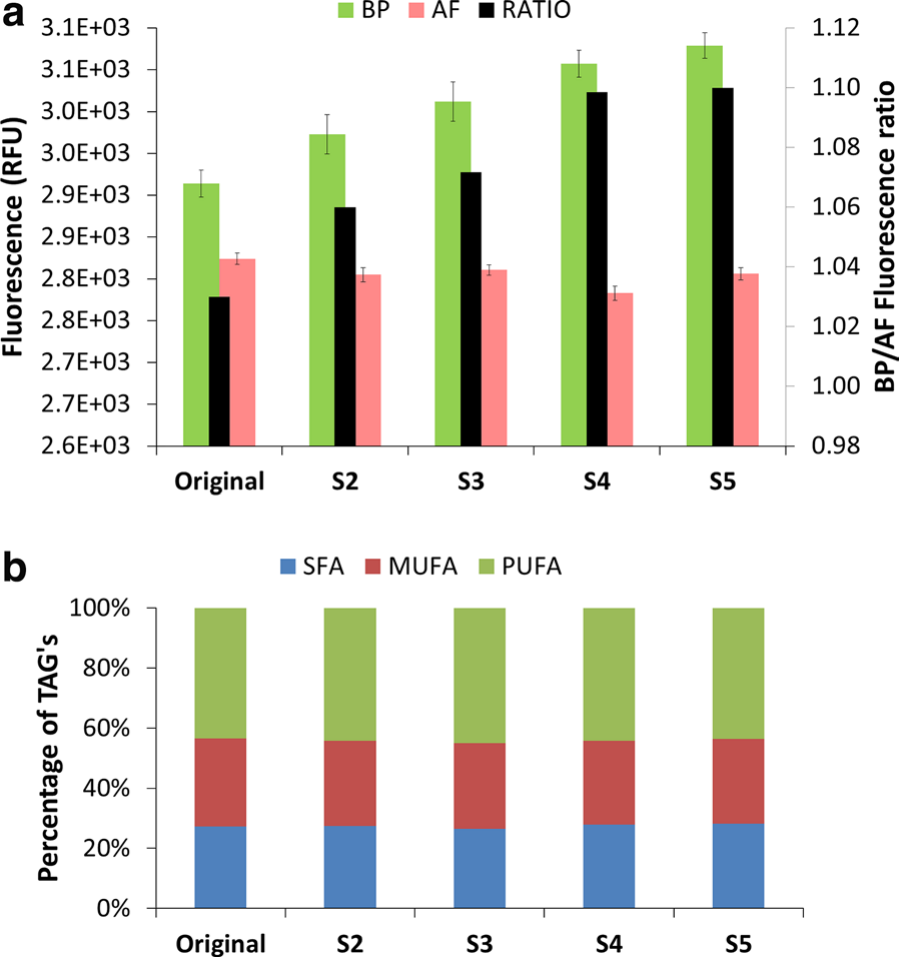
A progressive increase in lipid fluorescence leads to a progressive increase in BP/AF ratio without changes in the saturation degree of TAG’s. Fluorescence measurements of both Bodipy (BP) and autofluorescence (AF) on the primary *y*-axis and the BP/AF ratio on the secondary *y*-axis in all populations (*x*-axis) (**a**). All populations showed similarities in the TAG’s saturation degree (SFA, MUFA and PUFA’s as  % of total TAG’s) (**b**)

Original was run in parallel with S2–S5 to compare growth rates, biomass and lipid productivities and biomass composition. Figure 2 shows the overlapped growth curves for all five populations with a clear similarity among all populations during the growth phase (during the first 2 days N was available, green area). Table 1 gives the values of specific growth rates (µ) and biomass productivities (*P*_*X*_) among all sorted populations, which were similar to each other. Since cells were sorted based on their lipid content per cell size, the first parameter to be investigated was the relative lipid accumulation rate (BP_*r*_, day^−1^), showing that all sorted populations presented higher accumulation of lipids in comparison with the original (1.15–1.20 against 1.09 day^−1^, Table 1). Next, the median fluorescence values (both AF and BP) were compared, revealing an progressive increase in median BP fluorescence while the AF was reduced (Fig. 3a). This resulted in a progressive increase in the BP/AF ratio, the aim of our sorting criteria. The parameter BP/cell was calculated dividing the median value of BP-fluorescence by the total cell number (at the end of the N-runout, thus at day 8), resulting in a similar trend as the BP_*r*_. These parameters were used to decide to continue/stop the sorting in the early stage of the research as an indication of the lipid productivity. It should be highlighted that they are relative measurements, hence not capable of replacing the actual measurement of lipid productivity, which can only be determined after sorting a new population when enough biomass is produced. Biomass samples of all populations were taken at the end of the comparison runs to measure the TAG productivity.

**Table 1:** Comparing kinetic parameters and biomass composition between original and sorted populations

Kinetic parameters	µ (day^−1^)	*P*_*x*_ (g l^−1^ day^−1^)	P_TAG_ (g l^−1^ day^−1^)	BP_*r*_ (day^−1^)	BP/cell (RFU cell^−1^)
Original	0.69	1.54	0.18	1.09	1.09
S2	0.67	1.48	0.20	1.15	1.12
S3	0.73	1.66	0.20	1.22	1.14
S4	0.64	1.39	0.25	1.19	1.20
S5	0.67	1.48	0.34	1.20	1.23

Biomass composition	Starch (g g^−1^)	Carbs (g g^−1^)	Proteins (g g^−1^)	PL (g g^−1^)	TAG (g g^−1^)
Original	0.24	0.10	0.14	0.05	0.23
S2	0.24	0.12	0.15	0.06	0.27
S3	0.25	0.12	0.14	0.05	0.26
S4	0.24	0.13	0.16	0.06	0.29
S5	0.25	0.12	0.16	0.06	0.34

The kinetic parameters are: specific growth rate (µ) and biomass productivity (*P*_*x*_), both under growth conditions (between *t* = 0 and *t* = 2). TAG productivity (P_tag_) and lipid fluorescence accumulation rate (BP_r_) consider the whole N-starvation period (*t* = 2 to *t* = 8), while lipid fluorescence per cell was measured considering the values at the end of the starvation phase (*t* = 8). All analyzed biomass components are expressed as relative to biomass dry weight (g g^−1^ DW^−1^). Standard variation of measurements is not depicted because the technical error was always around 5 %

TAG productivity (P_TAG_) was measured for all populations, showing increased P_TAG_ values among all sorted populations (Table 1). Compared to original, S2 and S3 showed a discrete increase (from 0.18 in original to 0.20 g l^−1^ day^−1^ in S2 and S3) and S4 showed an increase of 38 % (from 0.18 to 0.25 g l^−1^ day^−1^). S5 showed the highest P_TAG0_, resulting in an 88 % increase in P_TAG_ when compared with original (from 0.18 to 0.34 g l^−1^ day^−1^). The biomass produced by all sorted populations was analyzed for its content as total starch, total carbohydrates (minus starch), total proteins, polar lipids and TAGs (Table 1). The biomass composition of all sorted populations and the original population was similar among each other, with the exception of TAGs. The analyses of biomass composition showed that the extra biomass produced by the sorted populations was caused only by an overproduction of TAGs (Table 1).

We would like to highlight that the sorted criteria used in the current research didn’t affect the TAGs composition of all sorted populations, which showed the same saturation degree (Fig. 3b). This finding was intentional and expected, considering the staining used in the current research. Bodipy (505/515) is a lipophilic fluorophore that binds to neutral lipids, being a relative measurement of total TAGs [26, 27]. Hence, in our work cells were selected based on total TAGs content per cell, without applying any pressure that would favor a change in TAGs composition. Two previous works on microalgae strain improvement with cell sorting have reported a change in lipid composition [7, 10]. Both works, however, have used random mutagenesis, which could explain the change in lipid composition.

**Fig. 4:**
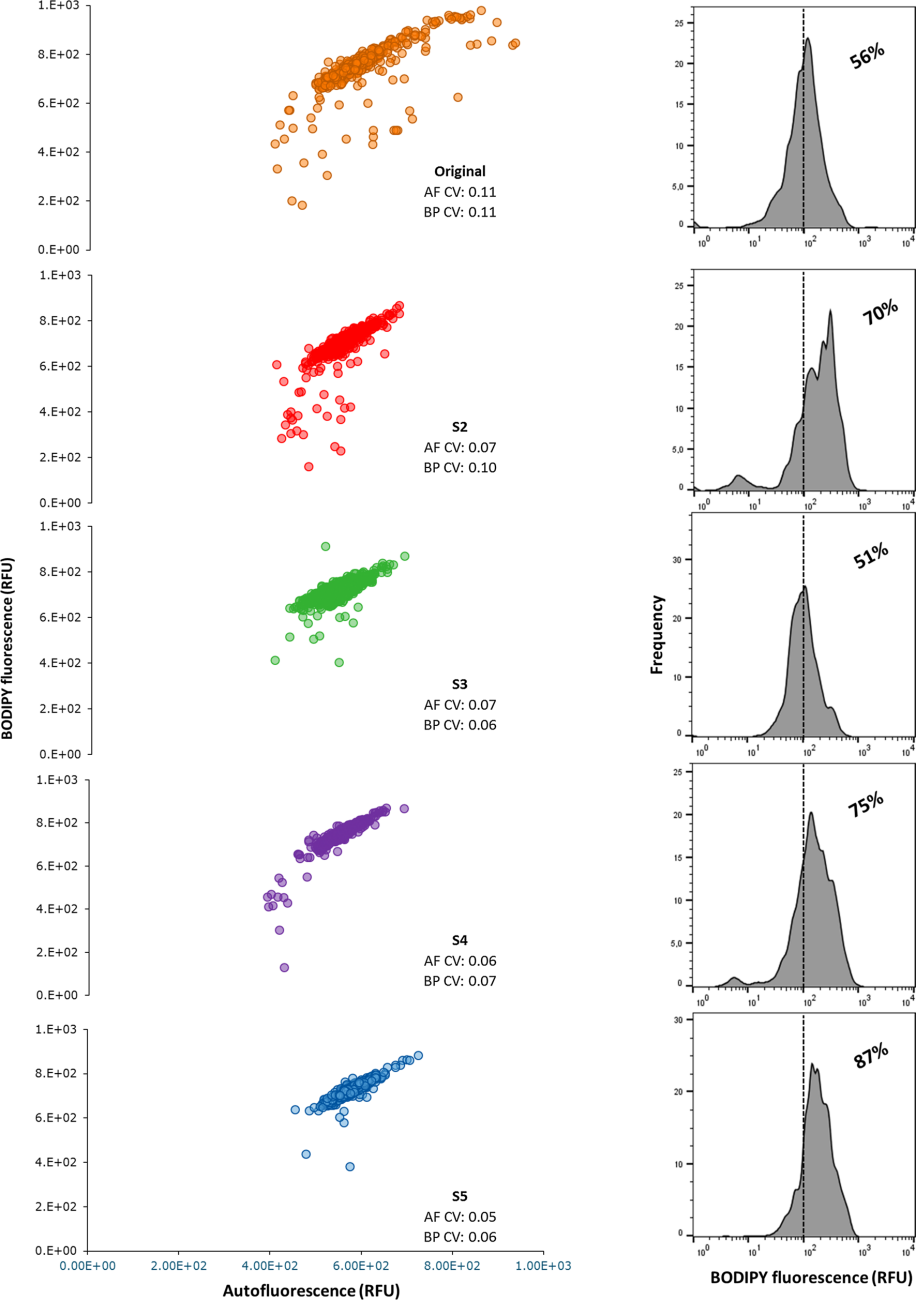
Sorted populations show a progressive increase in lipid productivity due to an increase in the proportion of lipid-rich cells within the populations. *Scatter plots* represent the relation between autofluorescence (AF, *x*-axis) and lipid fluorescence (BP, *y*-axis). On all *scatter-plots* the coefficient of variation (CV) of both AF and BP are depicted. Each populations has a histogram plotted showing the frequency distribution of lipid fluorescence (BP, *x*-axis). Additionally, each histogram is market to show the percentage of cells that have fluorescence signal above 10^2. All graphs represent the readings of 1000 cells

Flow cytometry data were used to see the relationship at cellular level, between relative lipid content and autofluorescence (Fig. 4, scatter plots). From such relation we can see that the sorted cells don’t show fluorescence values above the maximum already exhibited by the Wt. We hypothesized that our sorted cells would achieve higher values of BP-fluorescence than the Wt, similar to what has been reported by Thi-Thai Yen Doan and Obbard [8] and by Montero et al. [13]. What we see, however, is that we increased the lipid/cell ratio by progressively removing the cells with a low lipid/cell ratio, hence increasing the median BP fluorescence of the population. This effect can be visualized in numbers using the coefficient of variation (CV) of both auto and BP-fluorescence, which were progressively reduced in all sorted populations (values available on the scatter plots, from 0.11 (Wt) to 0.06 (S5), Fig. 4). The results from the scatter plots (Fig. 4) can be combined with the histograms of frequency of BP (right side, Fig. 4). The histograms show that the populations have a higher median TAG content because they show a higher proportion of the population composed of lipid-rich cells (percentage values on the charts show the proportion of cells within the population that have fluorescence values above 10^2^ RFUs). We can conclude, combining all results above, that the sorting carried out in the present research was successful in producing new cell lines with increased lipid productivity.

Previous research articles have also reported successful sorting of microalgae cells to produce populations with increased lipid content [7, 8, 10, 13, 16]. All these publications, however, do not measure the impact of the sorting on biomass or lipid productivity. Furthermore, all these publications were done at small laboratory scale, many using well-plates or shake flasks, hence the estimation of industrial performance of strains is limited. One exception was the work of Beacham and co-authors, which assessed both growth rate and lipid productivity of *Nannochloropsis salina* and sorted mutants [15]. The combination of mutagenesis and cells sorting lead to 4 mutants, all exhibiting an overproduction of lipids, but all mutants also exhibited a reduction in biomass productivity (from −18 to −95 % growth). In their results only one mutant showed a final lipid productivity higher than the original strain (from 0.40 to 0.49 × 10^−4^ g ml^−1^ day^−1^).

Another important remark when comparing sorted cells/mutants is the timing in between sorting rounds, which may cause an effect on growth and lipid kinetics of sorted populations. Most of other works above mentioned compared the lipid content of cell populations cultivated immediately after being sorted, hence ignoring a possible effect of the post-sorting period of re-growth on the physiological response of microalgae [7, 8, 14–16]. Previous research has found a 3.2 times longer growth phase in sorted populations in comparison with the original [14]. Since the growth was measured immediately after sorting, it is impossible to say if the slower growth was a feature of the new population or a longer lag phase caused by the sorting process. The effects of the sorting process itself could decrease growth rate and/or affect the lipid metabolism response since it represents a source of stress and the cellular response is strain-specific [17, 28, 29]. The above discussed results highlight the importance of assessing cellular growth after a long period post-sorting, to guarantee the stability of the sorted population to industrial cultivation.

### Comparing original and S5 under simulated Dutch summer conditions

The parallel runs with sorted populations and original population showed S5 as the population with the highest TAG productivity. The next step was to cultivate original and S5 under simulated outdoor conditions. This is an important step since part of the biomass (carbons sources, e.g. starch and TAGs) can be respired during dark periods for cellular maintenance, leading to possible changes in the productivity of different biomass components when comparing to experiments under continuous light [30]. The goal of this comparison was to evaluate how superior the productivity of S5 was in comparison with original under day/night cycles, hence we simulated an average Dutch summer day in an indoor flat panel reactor.

We also used the comparison experiment between S5 and original to estimate the stability of the sorted phenotype as the comparison experiments were carried out 1.5 years after the population had been sorted. Original and S5 were kept under low light in agar plates containing growth medium in the time between the sorting and the comparison experiments (cultures were transferred to new plates every 3 months). Therefore, original and S5 were compared using inoculum that came from similar conditions, and both were done in biological replicates, all combined to make the comparison more accurate.

**Fig. 5:**
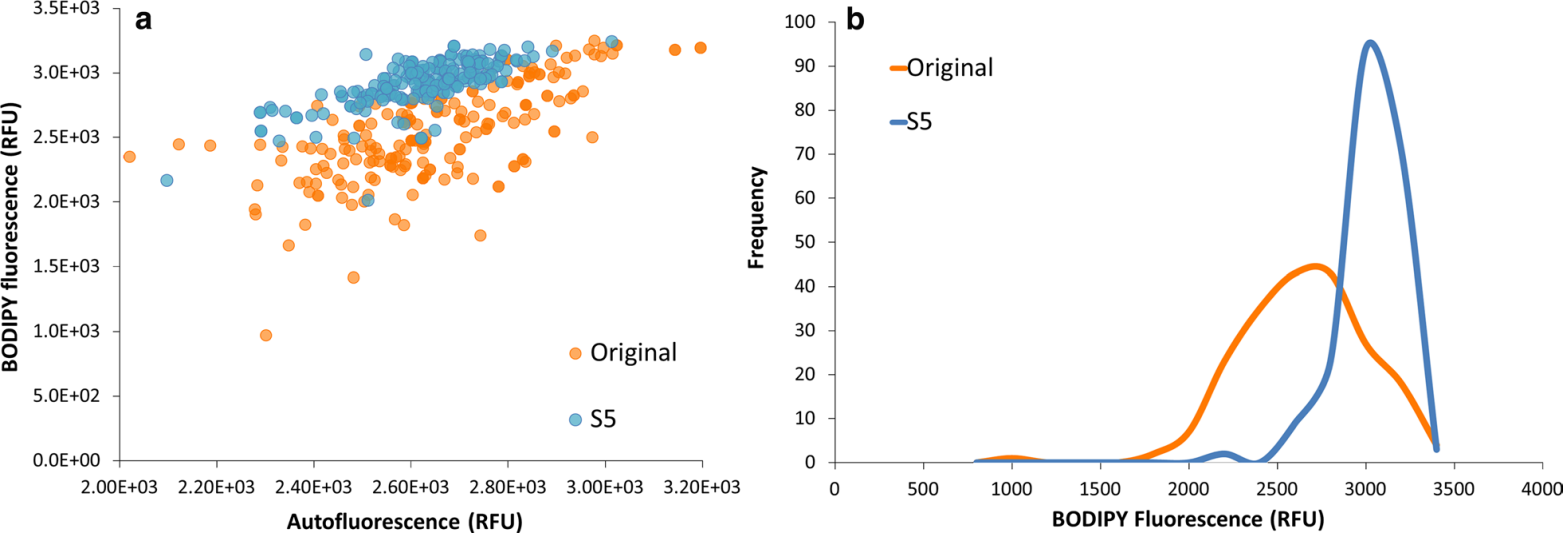
Comparing growth and lipid accumulation of original and S5 under simulated Dutch summer conditions. *Graph* **a** shows that the S5 strain produces more biomass under nitrogen starvation than the Wt. The *grey* area represents the period of the cultivation in which N was available, hence both populations consumed nitrogen at the same rate. From day 2 onward the N-starvation phase (N−) is considered*. Error bars* are depicted at each time point (standard deviation, three replicates), although for most of the points the* bars* are too small to be visualized. *Graph* **b** shows the difference between S5 and original at cellular level (at the end of the N-starvation period): the S5 population has higher values of Bodipy fluorescence than the Wt, leading to an increase in the BP/AF ratio. Both populations had the same number of cells analyzed (*n* = 500). *Error bars* at figure **b** represent the 95 % confidence intervals

Figure 5a shows the growth (as biomass DW, g l^−1^) of original and S5, confirming our results from the parallel runs: under growth conditions (days 0–2, Fig. 5) S5 shows similar growth kinetics as the original (µ and *P*x, Table 2). Previous research articles have reported that cells with improved lipid content presented also a reduction in growth [15, 10]. Our results, however, highlight that the sorting criteria used in the current work didn’t affect cell growth. One explanation could be the inclusion of AF as part of the selection criterion. We hypothesized that cells that could keep their levels of AF after a period of N-starvation would be cells with low levels of chlorophyll degradation, hence cells that could grow again if N is re-supplied.

**Table 2:** Comparing kinetic parameters and biomass composition between original and S5 under indoor simulated Dutch summer conditions

Populations	µ (day^−1^)	P_*X*_ (g l^−1^ day^−1^)	P_TAG_ (g l^−1^ d^−1^)	Y_TAG_ (g mol^−1^)
Original	0.52 ± 0.08^a^	1.03 ± 0.19^a^	0.2 ± 0.02^a^	0.16 ± 0.01^a^
S5	0.48 ± 0.03^a^	1.08 ± 0.08^a^	0.4 ± 0.06^b^	0.32 ± 0.03^b^

	Starch (g g^−1^)	Carbs (g g^−1^)	Polar lipids (g g^−1^)	TAG (g g^−1^)
Original	0.25 ± 0.04^a^	0.20 ± 0.04^a^	0.07 ± 0.01^a^	0.21 ± 0.02^a^
S5	0.29 ± 0.01^a^	0.19 ± 0.05^a^	0.06 ± 0.01^a^	0.30 ± 0.04^b^

The kinetic parameters are: specific growth rate (µ), biomass productivity (*P*_*x*_), TAG productivity (P_tag_) and TAG yield (Y_TAG_). All analyzed biomass components are expressed as relative to biomass dry weight (g g^−1^ DW^−1^). Error bars in the table indicate the standard deviation. Superscript letters represent the results of the statistical analyses between Original and S5. Different letters indicate statistical significance (ANOVA, *p* < 0.05)

**Fig. 6:**
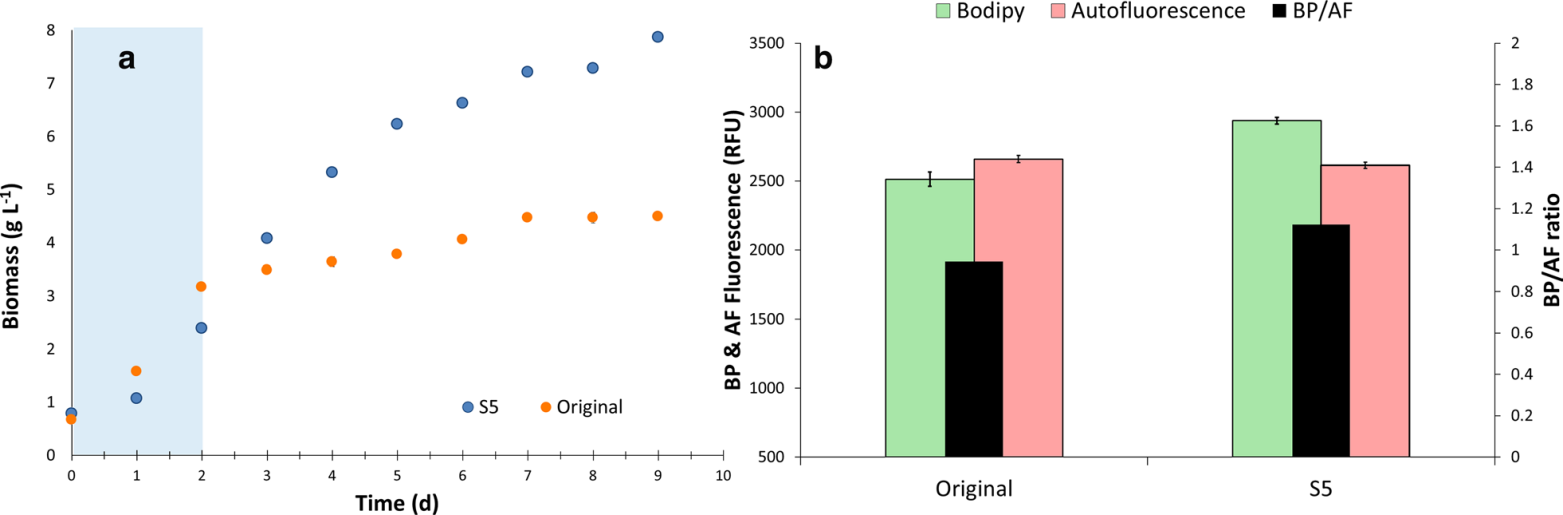
S5 population has a higher percentage of lipid-rich cells than the original population. *Graph* **a** shows a *scatter-plot* between autofluorescence and Bodipy fluorescence at the end of the N-runout. Each *dot* represents one-cell measurement. *Graph* **b** shows the distribution of frequency of Bodipy-fluorescence of both original and S5. In both *graphs* a total number of 500 cells were analyzed per sample

Differences, however, were observed after the start of the N-runout (Fig. 5, after day 2). Biomass composition shows again that TAGs were the biochemical fraction that explained the increase in biomass accumulation in the N-starvation period (Table 2). The TAG content of S5 was 1.42 × higher than original (0.30 against 0.21 g/g DW, Table 2), while other biomass components (starch, carbohydrates (minus starch) and polar lipids) were similar between original and S5. The increase in TAG content resulted in a twofold increase in the P_TAG_ of S5 when compared with original (Table 2), thus confirming the results of the first experiments. Cytometry data were used to evaluate the cause for increase in lipid productivity at cellular level. Flow cytometry data from the cells at the end of the cultivation corroborate the previous results since we observe once again an increased median lipid fluorescence (BP) being the responsible variable that results in an increased BP/AF ratio (Fig. 5b). Additionally, Fig. 6 shows that S5 has a higher percentage of lipid-rich cells under similar conditions when compared with Wt. The amount of lipid-rich cells in S5 was 99 % while in the original population it was 66 % (arbitrarily chosen as cells with fluorescence signals above the half of the scale; numbers derived from the histograms on Fig. 6). Added to that, biomass composition indicates that S5 has no alterations in the carbon partitioning between TAG and starch (Tables 1, 2), but only an doubled TAG yield on light when compared to the original population. Hence, the strategy here presented was successful in selecting microalgae cells with increased TAG yield without affecting the metabolism of other cellular components.

The results from our experiments were compared with other researches with batch-wise TAG production. A review of TAG production is given by Benvenuti et al. [31], comparing different microalgae strains using the yields of TAG on light to compensate for different growth/stress conditions. We calculated the TAG yields on light of both original and S5 as being 0.16 and 0.32 g mol^−1^, respectively (under simulated summer conditions). First it is important to highlight that original *C. littorale* showed an TAG yield close to other high-lipid microalgae, such as: *Scenedesmus obliquus*; 0.22 g mol^−1^; *Nannochloropsis oculat*a, 0.17 g mol^−1^; *Chlorella zofingiensis*, 0.19 g mol^−1^ and *Nannochloropsis* sp., 0.14 g mol^−1^ [32–35]. The work of Breuer et al. [33] also presents the TAG yield of an starchless mutant of *S. obliquus* at 0.37 g mol^−1^, close to the yields measured for the S5 in the current work (0.32 g mol^−1^).

## Conclusions

In this work, we presented an approach to select microalgae cells with increased lipid productivity under N-starvation. Our approach was successful because we successfully selected new cell lines with increased lipid content (under N-starvation) with no effect on growth (N supplied), resulting in progressive improved TAG productivities. S5, sorted after 5 cycles of starvation-sorting, showed 1.9× higher TAG productivity than the Wt. Hence, S5 and original were compared under simulated summer conditions in a flat-panel photobioreactor. The results from comparing S5 and original confirmed the results of the first experiment: S5 showed an 2× higher TAG productivity (from 0.2 to 0.4 g l^−1^ day^−1^) because we have removed cells with low TAG yield (on light) in comparison to Wt. The experiments comparing S5 and original were done 1.5 year after the sorting, therefore, indicating a stable phenotype. S5 showed superior TAG yields on light when compared with other Wt-high-lipid green microalgae (0.32 against 0.16–0.20 g mol^−1^), being comparable to the highest yield registered with a starchless mutant (0.32 against 0.37 g mol^−1^). Biomass composition indicates that S5 has no alterations in the carbon partitioning between TAG and starch, but only an doubled TAG yield on light when compared to the original population. All combined, our results present a successful strategy to improve the TAG productivity of *C. littorale*, without resourcing to genetic manipulation or random mutagenesis. Additionally, the improved TAG productivity of S5 was confirmed under simulated summer conditions, highlighting the industrial potential of S5 for microalgal TAG production.

## Abbreviations

µ: growth rate (per day)
AF: aufluorescence (chlorophyll related)
BP: bodipy
BP_*r*_: bodipy accumulation rate (per day)
Carbs: other carbohydrates not including starch
CI: confidence intervals
CV: coefficient of variation
FACS: fluorescence assisted cell sorting
N: nitrogen
NHST: null hypothesis statistical testing
P_TAG_: TAG volumetric productivity (g l^−1^ day^−1^)
P_*x*_: biomass volumetric productivity (g l^−1^ day^−1^)
TAG: triacylglycerols
Y_TAG_: TAG yield on photons (g mol^−1^)
